# Diversity and Biosynthetic Potential of Fungi Isolated from St. John’s Island, Singapore

**DOI:** 10.3390/ijms24021033

**Published:** 2023-01-05

**Authors:** Madhaiyan Munusamy, Kenneth Tan, Choy Eng Nge, Martin Muthee Gakuubi, Sharon Crasta, Yoganathan Kanagasundaram, Siew Bee Ng

**Affiliations:** 1 Singapore Institute of Food and Biotechnology Innovation (SIFBI), Agency for Science, Technology and Research (A*STAR), 31 Biopolis Way, #01-02 Nanos, Singapore 138669, Singapore; 2 School of Biological Sciences, Nanyang Technological University, Singapore 637551, Singapore

**Keywords:** antimicrobial activity, biosynthetic gene clusters, nonribosomal peptide synthetases, polyketide synthases

## Abstract

Adaptation to a wide variety of habitats allows fungi to develop unique abilities to produce diverse secondary metabolites with diverse bioactivities. In this study, 30 *Ascomycetes* fungi isolated from St. John’s Island, Singapore were investigated for their general biosynthetic potential and their ability to produce antimicrobial secondary metabolites (SMs). All the 30 fungal isolates belong to the Phylum *Ascomycota* and are distributed into 6 orders and 18 genera with Order *Hypocreales* having the highest number of representative (37%). Screening for polyketide synthase (PKS) and nonribosomal peptide synthetase (NRPS) genes using degenerate PCR led to the identification of 23 polyketide synthases (PKSs) and 5 nonribosomal peptide synthetases (NRPSs) grouped into nine distinct clades based on their reduction capabilities. Some of the identified PKSs genes share high similarities between species and known reference genes, suggesting the possibility of conserved biosynthesis of closely related compounds from different fungi. Fungal extracts were tested for their antimicrobial activity against *S. aureus*, Methicillin-resistant *S. aureus* (MRSA), and *Candida albicans*. Bioassay-guided fractionation of the active constituents from two promising isolates resulted in the isolation of seven compounds: Penilumamides A, D, and E from strain F4335 and xanthomegnin, viomellein, pretrichodermamide C and vioxanthin from strain F7180. Vioxanthin exhibited the best antibacterial activity with IC_50_ values of 3.0 μM and 1.6 μM against *S. aureus* and MRSA respectively. Viomellein revealed weak antiproliferative activity against A549 cells with an IC_50_ of 42 μM. The results from this study give valuable insights into the diversity and biosynthetic potential of fungi from this unique habitat and forms a background for an in-depth analysis of the biosynthetic capability of selected strains of interest with the aim of discovering novel fungal natural products.

## 1. Introduction

The natural environment harbors a wide variety of microorganisms representing an enormous reservoir of secondary metabolites with untapped chemical potential. Fungi represent one of the largest and most diverse groups of living organisms [1]. However, although 5 to 6 million species of fungi are estimated to exist, only a small fraction of these microbes has been characterized [2,3]. Moreover, an even smaller fraction of the characterized fungal strains has been studied for their natural products’ biosynthetic potential. Recently, there has been an increased interest in exploration of the Fungi kingdom for natural products due to the huge variety of species within this kingdom and the diverse habitats in which they are found [4,5,6]. The ability of fungi to colonize a wide range of habitats—from terrestrial to marine environments—grants them unique adaptive capabilities which underscore their potential to produce a vast repertoire of natural products with potential pharmaceutical and biotechnological applications [7,8,9,10,11].

The onset of the genomic era followed by the exponential rise in availability of genomic data has led to the development of computational tools for genome mining capable of identifying biosynthetic gene clusters (BGCs) involved in the fungal secondary metabolism [12,13,14]. However, there is a noticeable disparity between the number of BGCs predicted by genome mining and the actual number of known compounds identified for any given microbial strain [15,16,17,18]. For instance, a study analyzing *Penicillium* genomes found 798 BGCs, but only 16% of the these could be connected to a pathway with a known product [19]. 

A separate study of fungal BGCs reported in the literature found a disproportionate representation by certain fungal genera [20]. Furthermore, the vast majority of BGCs characterized with known chemical products come from only three fungal genera: *Aspergillus*, *Fusarium*, and *Penicillium*, suggesting that there is much chemical diversity which remains to be discovered [20].

Within fungal natural product research, considerable attention is given to the discovery of antimicrobials due to the threat of emerging multidrug resistant pathogens that reduce the effectiveness of current antibiotics against life-threatening bacterial and fungal infections [21,22]. In the last decade, many novel bioactive natural products from fungi with antibacterial and antifungal activities have been discovered; prompting a surge of interest in the discovery of fungal strains with interesting chemical profiles [16,23,24]. Bioactivity-guided screening has traditionally been used for drug discovery efforts as it allows for the isolation of the active constituents, relative to untargeted metabolomics which face challenges in differentiating LC-MS signals of secondary metabolites from other molecules produced by the host organism [15,25]. Specifically, bioassay-guided fractionation and isolation of compounds have contributed to the discovery of a wide range of active compounds with antimicrobial potential [26,27,28].

The two major classes of natural products synthesized by fungi are polyketides and nonribosomal peptides [29,30]. They include toxins, pigments, siderophores, antibiotics, and immunosuppressants among other biologically active molecules [30,31,32,33]. In this study, we report a screening campaign of 30 fungal strains isolated from terrestrial and marine habitats on St. John’s Island, Singapore, with the aim of exploring their biosynthetic and antimicrobial potential. To evaluate the genetic potential of the fungal isolates to synthesize these classes of compounds, we have used degenerate primer sets which recognize polyketide synthases (PKSs) and nonribosomal peptide synthetases (NRPSs) to screen for these signature genes by PCR. Moreover, in an effort to discover active antimicrobial compounds from fungi, we have used bioassay-guided fractionation and LC-MS to identify bioactive compounds.

## 2. Results

### 2.1. Phylogenetic Diversity of Fungal Isolated Strain

**Figure 1:**
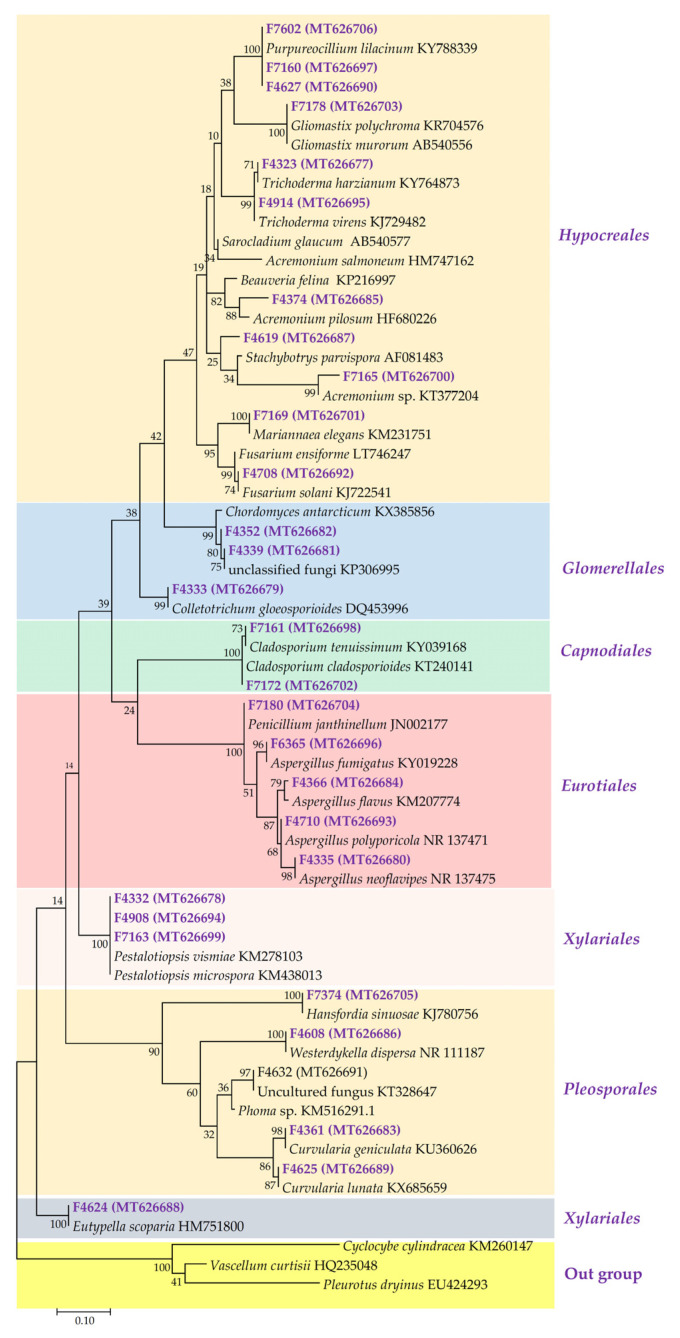
The phylogenetic tree of the 30 ascomycetes (shown in purple) constructed using ITS2 sequences. The tree topology is based on the neighbor-joining method. ITS2 sequences from three *Basidiomycota* species were used as outgroups. Reference sequences obtained from GenBank have their accession numbers indicated. Bootstrap values greater than 50% are shown at the nodes. The tree is drawn to scale; scale bar represents nucleotide substitutions per site. Evolutionary analyses were conducted in MEGA7. Accession numbers are shown in parentheses.

Taxonomic analysis of the 30 isolated fungal strains was performed by examining the sequences of their ITS2 region and comparing this with the sequences uploaded onto the National Center for Biotechnology Information (NCBI) Basic Local Alignment Search Tool (BLAST; https://blast.ncbi.nlm.nih.gov/Blast.cgi, accessed on 29 October 2022) to find similar sequences. All the 30 fungal isolates belong to the Phylum *Ascomycota* and are distributed into 6 orders and 18 genera (Figure 1) with Order *Hypocreales* having the highest number of representative (37%). The most prevalent genera include *Aspergillus* (four strains), *Pestalotiopsis* (three strains), and *Purpureocillium* (three strains). Certain strains (F4632 and F4619) had incomplete taxonomic information as the best BLASTN hits were returned as uncultured/environmental sample sequences which are uncharacterized. Strains F4632 and F7165 for instance were identified only up to the Genus level; namely, *Phoma* sp. and *Acremonium* sp., respectively, because the top hits from BLASTN for the two isolates were uncultured environmental isolates. F4374 also appeared to form a distinct clade away from its nearest relatives, suggesting that it might be a potential novel strain, although further phylogenetic studies may be necessary to ascertain this. Interestingly, F4339 and F7165 aligned strongly (100% and 95% identity, respectively) with the ITS sequence entries from a particular study of sponge-associated fungi from the Caribbean and Pacific of the Republic of Panama [34], suggesting that tropical habitats may host a rich repertoire of uncharacterized fungal species. Indeed, some of our strains have no nomenclatural status and have only recently been given a new genus. F7374 was identified as *Ascotricha sinuosa*, a newly described endophytic fungi of the marine brown alga *Colpomenia sinuosa* [35]; it was previously classified as *Hansfordia sinuosa* [36]. F4339 and F4352 matched to *Hypocreales* and *Acremonium* sp. according to several hits on BLASTN. However, when placed on the phylogenetic tree (Figure 1), they appear to form a new clade which grouped them together with *Chordomyces antarcticum*, a newly designated alkalitolerant species of fungi [37].

### 2.2. Presence of PKS and NRPS Genes and Its Phylogeny

Degenerate PCR was used to screen for putative PKS and NRPS genes from the fungal genomic DNA. Because fungal genomes often contain multiple PKS genes, we sought to probe for and distinguish between the different PKS subclasses based on the extent of reduction of the polyketide [38,39]. This enables a more thorough determination of each strain’s biosynthetic potential. For detection of NRPS, we chose primers AUG003 and AUG007 as they target the A and T domains [40] and have been used on related studies focusing on marine fungi biosynthetic potential [8,41]. Primers specific to only A domain (such as primers RJ016-F and RJ016-R) gave PCR product sizes of just 300 bp, and instead resulted in sequences displaying greater similarity to bacterial NRPSs in reported phylogenetic trees [42].

In the current study, 23 PKS (12 non-reducing PKS, eight partially reducing PKS, and three highly reducing PKS) and five NRPS genes were identified from 18 fungal strains (Table 1). The gene fragments had a nucleotide length of between 630 to 791 bp for PKS, and around 1200 bp for NRPS (Table S1). BLASTN analysis showed that 10 DNA sequences did not have significant similarities matches from NCBI (Table S1), indicating that the sequences might be unique; at least at the nucleotide level. BLASTX (translated nucleotide search) was used to match each gene fragment to the closest annotated protein from NCBI (Table 1), and to reveal conserved domains confirming the product identity (Table S1). PKS sequences possessed between 68% and 100% amino acid identity to known fungal PKSs, while NRPS amino acid sequence identities ranged between 40% and 99% (Table 1). The majority of the PKS genes belong to the non-reducing (NR) PKS, 12 genes (six from terrestrial and six from marine isolates), followed by partially reducing (PR) PKS, eight genes (four from terrestrial, four from marine isolates). For the highly reducing (HR) PKS, three genes (two from terrestrial, one marine isolates) were uncovered while NRPS genes were only found in four terrestrial and one marine fungal isolate (Table 2).

**Figure 2:**
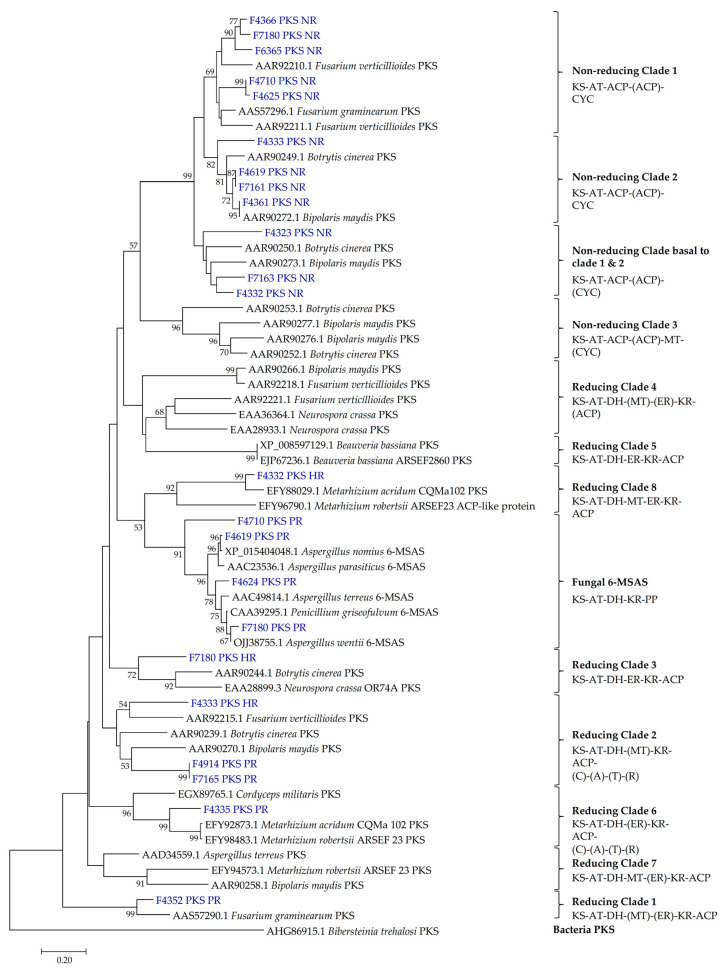
The phylogenetic tree of the ketosynthase domains of fungal polyketide synthases (PKSs) detected in this study (shown in blue). Reference PKS sequences of known clades were obtained from previous reports [43,44]. The common organization of domains is indicated with the clades. Domains in parentheses are variable in their presence/absence within the respective clades. GenBank accession numbers are shown beside each reference entry. A bacteria PKS from *Bibersteinia trehalosi* was used as an outgroup. Evolutionary relationships were determined by the neighbor-joining method, and bootstrap values of ≥50% are shown above the nodes. The scale bar represents 0.2 amino acid changes.

**Figure 3:**
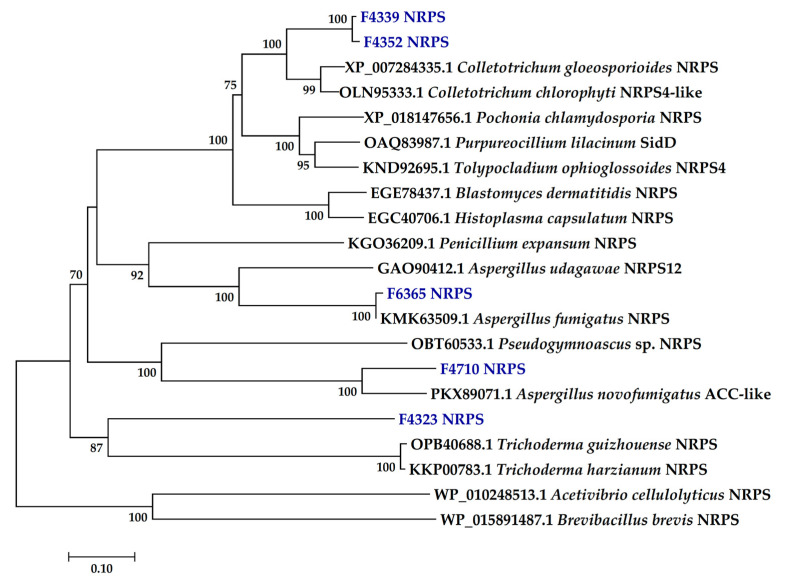
The phylogenetic tree of fungal nonribosomal peptide synthetases (NRPS) detected in this study (shown in blue). GenBank accession numbers are shown beside each reference entry. NRPSs from two bacteria; *Acetivibrio cellulolyticus and Brevibacillus brevis* were used as outgroups. Evolutionary relationships were determined by the neighbor-joining method, and bootstrap values of ≥50% are shown above the nodes. The scale bar represents 0.1 amino acid changes.

Phylogenetic analysis was performed for PKS and NRPS sequences (Figure 2 and Figure 3). The diversity of PKSs can be visualized by classifying them according to reference fungal PKSs of known clades [43,44]. The most recent classification system constitutes four clades of non-reducing (NR) PKSs and eight clades of reducing PKSs (Figure 2). PKSs from this study were well distributed across nine clades: NR clade I (five genes), NR clade II (four), NR basal to clades I and II (three), reducing clade I (one), reducing clade II (three), reducing clade III (one), reducing clade VI (one), reducing clade VIII (one), and fungal 6-methylsalicylic acid synthase (6-MSAS) clade (four) (Figure 2). Genes belonging to the fungal NR PKS—which lack the dehydratase (DH), enoyl reductase (ER), and *β*-ketoacyl reductase (KR) domains—were distributed into a distinct clade from the reducing PKSs (Figure 2). Compared to the PR PKSs, HR PKSs were grouped into clades 3 and 8 (Figure 2), which are characterized by the presence of all three reducing domains (DH, ER, KR).

The PKS genes from highly related species were clustered together. For instance, four out of five genes in NR clade I were derived from strains of the order *Eurotiales* (*Aspergillus* and *Penicillium*). Surprisingly, the other contributor was F4625 (*Curvularia lunata*), whose NR PKS gene was closely matched on BLASTX with *Aspergillus terreus* PKS (Table 1), similar to the NR PKS of F4710 (*Aspergillus polyporicola*). Furthermore, four of our strains had PKS belonging to the 6-MSAS-type, a group of fungal iterative PKS hypothesized to have been obtained from bacteria via ancient horizontal gene transfer [45]. The four strains belong to the Genus *Aspergillus*, *Eutypella*, *Penicillium*, and *Stachybotrys*.

An intriguing class of PKS—the PKS-NRPS hybrids—can be clustered into two clades: Reducing clade II and reducing clade VI (Figure 2). The PKS genes in these clades contain additional NRPS domains (condensation, adenylation, thiolation, and reductase domain) which enable them to develop different functional activities from the typical PKS and NRPS gene clusters [46]. Characterized proteins include the *A. terreus* LovB and *Penicillium citrinum* MlcA, which synthesize the cyclic nonaketide portion of lovastatin and citrinin, respectively [43]. Four amplicons from this study were represented in reducing clades II and VI (Figure 2). Notably, the PKS sequences of F4914 and F7165 strains, both of which matched a putative *Trichoderma virens* PKS-NRPS protein (Table 1), were clustered with strong bootstrap support (99%) within the reducing clade II, suggesting that their gene products could be involved in the biosynthesis of the same or closely related compounds (Figure 2). Finally, F4335 PKS fell into reducing clade VI, representing a minor group of uncommon PKS-NRPS enzymes that were hypothesized to have evolved from reducing clade II [44]. Five NRPS genes were detected from F4323, F4339, F4352, F4710, and F6365, with most of the sequences displaying high bootstrap support to reference genes (Figure 3). Of these, only F6365 (*Aspergillus fumigatus*) NRPS had a 99% similarity to an annotated NCBI entry, while the closest match for F4339 and F4352 was a NRPS from *Colletotrichum chlorophyte* (Table 1). F4323 NRPS had the highest deviation from the closest reported reference, with a 67% similarity to an unannotated *Talaromyces islandicus* hypothetical protein (Table 1, Figure 3).

### 2.3. Antimicrobial Activity

**Figure 4:**
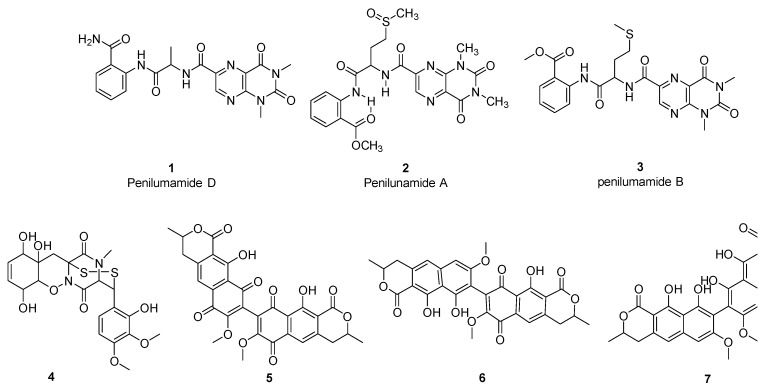
Structure of penilumamide D (**1**), penilumamide A (**2**), penilumamide B (**3**), pretrichodermamide C (**4**), xanthomegnin (**5**), viomellein (**6**), and vioxanthin (**7**).

Methanolic extracts from the 30 fungi strains were tested at a single concentration of 200 µg/mL against *S. aureus*, MRSA, and *C. albicans* with three strains; F4335, F4366, and F7180 presenting average growth inhibition ≥ 50% against at least one of the test microorganisms (Table 2 and Table S2). Two strains, F4335 and F7180 revealed promising antimicrobial activity and were progressed to bioassay-guided isolation of the bioactive compounds. This resulted in the isolation of three and four compounds from strain F4335 and F7180, respectively (Figure 4). The seven isolated compounds were subjected to antimicrobial testing against Gram-positive and Gram-negative bacteria, a yeast and cytotoxic activity against the human lung carcinoma cells, the result of which are summarized in Table 3. The three compounds identified from F4335—Penilumamides A, D, and E have been reportedly isolated from several *Aspergillus* species and were found to exhibit weak cytotoxicity activity against a human hepatoma cell line [47,48]. In the current study penilumamide A exhibited weak antibacterial activity against *S. aureus* with an IC_50_ value of 88 µM. Penilumamides A and B revealed weak cytotoxic activity against A549 cells with IC_50_ values 100 and 113 µM, respectively (Table 3). Strain F7180 produced a wide variety of bioactive compounds, namely xanthomegnin, viomellein, pretrichodermamide C, and vioxantin. All four compounds displayed moderate to strong antimicrobial activity against Gram-positive bacteria *S. aureus* and MRSA, with the best antibacterial activity observed for vioxantin, showing IC_50_ values of 3 µM and 1.6 µM against *S*. *aureus* and MRSA, respectively (Table 3). None of the seven compounds presented an IC_50_ < 50 µM against the Gram-negative bacteria *E. coli* (Table 3). With the exception of pretrichodermamide C, all the other compounds isolated from strain F7180 exhibited weak antifungal and cytotoxic activities against *C. albicans* and A549 cells, respectively (Table 3).

## 3. Discussion

Fungi are an understudied resource for the discovery of novel natural products [4,11,16,18,49,50,51]. Marine fungi account for more than 60% of the 456 newly reported marine microbial natural products [52], an indication of the huge scale of untapped biosynthetic potential of naturally occurring fungi. However, fungi are present in low diversity and abundance in many marine environments [53,54]. Terrestrial fungi are likewise an important grouping of fungi for the search of new secondary molecules especially because their molecular signatures have been simultaneously detected in deep-sea marine habitats, revealing a possible overlap in the metabolic capabilities of terrestrial and marine fungi [8]. To enhance the exploitation of fungi for biotechnological applications, it is essential to focus on unique and less studied habitats and environments for fungal isolation. In this study, we have assessed the diversity of fungal isolates from St. John’s Island, Singapore with the intention of exploring their biosynthetic and antimicrobial potential. St. John’s Island is home to diverse native and exotic plant species that are likely to harbor a unique community of fungal endophytes. Moreover, unlike mainland Singapore, the island has less human habitation and activities, thus providing a unique ecosystem for plants and associated microbiota.

### 3.1. Fungal Diversity

Most of the fungi from this study matched closely to related sequences in the NCBI database at 99–100% identity score on the basis of ITS sequencing analysis (Table S1). However, some of the isolates, such as strains F4339, F4374, F4619, and F7165 had a relatively lower homology (88% -97%) to annotated species’ sequences on NCBI and may be potentially novel and undescribed fungal strains (Table S1). On the contrary, these strains matched strongly with the ITS sequences of unidentified environmental isolates, for instance from sponge-associated fungi originating from the Caribbean and Panama suggesting that these species may require further taxonomic studies for correct phylogenetic placement [34]. In particular, although F4339 is a terrestrial isolate, its ITS sequence had a 100% homology to that of a marine fungal isolate (Accession no. KP306995.1) suggesting that the two might share a common ancestry [34,55] (Figure 1). Many studies on the phylogeny of marine fungi have also come to the same consensus that most marine fungi taxa are closely related to well-known terrestrial species [8,34,56,57]. This may suggest that such strains might have their origin in the terrestrial environment, which raises intriguing ecological questions regarding the ability of terrestrial fungi to tolerate and adapt to marine conditions including pressure, temperature, and salinity. This knowledge can be exploited for applications such as in the co-culture studies involving terrestrial and marine fungal strains to activate silent genes cluster and thus enhance the discovery of new natural products [56,58]. Phylogenetic diversity analysis showed that *Hypocreales* is the dominant order of fungi isolated in this study. Similar observations were reported in previous studies on marine fungi [34,41]. Several phylogenetic studies have shown that the majority of fungal endophytes belong to the phylum *Ascomycota* with a few representatives from other *phyla* such *Basidiomycota* and *Mucoromycota* (11, 16), and these findings are reflected in this study. Genera *Purpureocillium*, *Aspergillus*, and *Pestalotiopsis* were the most prevalent genera. New and less studied species such as the marine endophytic fungi *Ascotricha sinuosa* and *Eutypella scoparia* that were identified in the current study could be strains of interest for further detailed studies focusing on discovery of new bioactive compounds. Indeed, *Eutypella* species have attracted considerable interest in recent years and have been found to produce a diverse range of secondary metabolites, including pimarane diterpenoids, sesquiterpenoids, cytochalasin derivatives, polyketides, and *γ*-lactones [59,60,61,62].

### 3.2. PKS and NRPS

PKS and NRPS are appropriate targets for analysis of biosynthetic potential in fungi [8]. Our study utilized three pairs of degenerate primers which recognize NR, PR, and HR PKS genes respectively, and one pair of degenerate primers to detect NRPS genes. Eighteen out of 30 fungi strains (60%) showed the presence of either PKS or NRPS, a similar proportion to that cited in another study which found that PKS or NRPS accounted for 61% of the total BGCs in *Penicillium* genomes [19]. PKS genes (77%, 23/30) were detected at a higher frequently than NRPS genes (17%, 5/30), consistent with the findings of other studies focusing on ascomycetes biosynthetic potential [30,42]. Moreover, most of the PKSs identified had more than 80% homology with known KS genes while the majority of NRPS had less than 80% identity (Table 1). This difference could, however, be attributed to the regions amplified with the primers, since the PKS primers target the highly conserved KS domain while the NRPS primers target the adenylation and thiolation domains [63,64]. NRPS thiolation domains were found to have slightly less identities to known sequences as compared to the adenylation domains [65]. Nevertheless, lesser amino acid identities with the nearest known genes further imply that these genes could have the potential to synthesize novel natural products.

The results from this study show that approximately equal number of non-reducing (12 genes) and reducing PKSs (11 genes) were detected (Figure 2; Table 1). NR PKSs typically contain a Claisen-type cyclase (CYC), enabling them to synthesize cyclic (i.e., aromatic) unreduced polyketides including pigments such as melanin and bikaverin, but also precursors to toxins such as aflatoxin and sterigmatocystin [43]. On the other hand, reducing PKSs synthesizes mainly linear polyketides that serve as precursors for a wide range of compounds and toxins that are active in animals (e.g., lovastatin, citrinin, and fumonisin) and plants (e.g., T-toxin, PM-toxin) [43]. Gene products of PKSs in the reducing 6-MSAS clade are small monocyclic or polycyclic aromatic compounds, including food-contaminating mycotoxins such as patulin [66] and ochratoxin [67]. Reducing PKSs can increase the diversity of polyketide structures by generating reductions/dehydrations at specific positions along the chain using the ketoreductase (KR), dehydratase (DH), and enoyl reductase (ER) domains [68]. PKSs that lack some or all of these domains produce partially reduced or fully oxidized polyketides, therefore synthesizing polyketides with various chemical reductions in nature [43]. HR PKSs have all three reducing domains (DH, ER, KR), grouping them into reducing clades III and VIII (Figure 2). PR PKSs have DH and KR domains (Figure 2) and can be classified as 6-MSAS-type PKSs since most of them were involved in the synthesis of MSAS [38,68].

Depending on the additional domains they possess, PR PKSs can be grouped to reducing clades I, IV, V, and VII [44]. The polyphyly of PKS-NRPS hybrids is particularly interesting as they are clustered in two separate clades II and VI (Figure 2), with PKS-NRPS genes in clade VI hypothesized to have evolved from clade II [44]. Out of the 11 reducing PKS genes, four were clustered in the 6-MSAS clade and four were clustered in the PKS-NRPS hybrids clade. Recent studies have shown that fungal 6-MSAS and PKS-NRPS genes share a complex evolutionary history and may be of actinobacterial origin, as their clades nest deep inside bacterial PKS [43,69,70]. Growing evidence suggests that these genes could have been acquired through horizontal gene transfer events that happened early in the evolution of *Ascomycota*, thus bestowing upon the fungi enhanced biosynthetic capabilities [45,69].

Compared to the KS domains of PKSs, the majority of which had high sequence similarity to known reference genes, the NRPSs were more distant from their closest NCBI hits. In addition, unlike the PKSs, NRPS do not have clear clusterization to clades according to their structural diversity on the phylogenetic tree; a much larger sample size of NRPSs of different origin and function is needed to cluster them as mono/bimodular and multimodular enzymes [67,68,69,70,71]. The use of only one set of NRPS primers in our study may have restricted the number of NRPS detected because the primers may tend to amplify particular NRPS genes more efficiently than others, making genes with less conservation at the degenerate primer annealing sites difficult to clone. A combination of primers which recognize the different conserved NRPS domains might be needed to fully extend the range of detectable NRPS. For instance, degenerate primers matching the motifs A2, A5, A6, A7, and A8 could be used in addition to the A3 and T domains used here [40,72].

### 3.3. Bioactivity-Guided Fractionation and Isolation of Compounds

Two fungal strains, F4335 (*A. flavipes*) and F7180 (*P. janthinellum*) were selected for large-scale fermentation and compound isolation. We isolated penilumamide A, B, and D from F4335, and four different compounds from F7180 (xanthomegnin, viomellein, pretrichodermamide C, and vioxanthin).

Penilumamide, a new class of lumazine peptides recently isolated from *Aspergillus* and *Penicillium* sp., have been reported to be inactive against Gram-positive and Gram-negative bacteria, yeasts, and fungi [47,48,73]. These findings are largely consistent with our results, although we found that penilumamide A displayed weak antimicrobial and cytotoxic activity against *S. aureus* and A549 cells, respectively (Table 3). The Penilumamide biosynthetic gene cluster has not yet been characterized. However, it has been suggested that their biosynthesis may involve amino acid synthesis with the stepwise addition of methionine sulfoxide and anthranilic acid, followed by multiple C1 methylations, suggesting a possible role of NRPS in penilumamide biosynthesis [73]. Our genetic screen for PKS and NRPS (Table 2) identified a PR PKS from F4335, and its sequence was clustered in clade VI which represents a group of PKS-NRPS hybrids (Figure 2). However, BLASTX sequence identity with the closest NCBI entry is low (77% similarity with an *Endocarpon pusillum* hypothetical protein; Table 1), suggesting it may be a novel PKS-NRPS.

F7180 (*P. janthinellum*) produced xanthomegnin, viomellein, and vioxanthin, which are polyketide-derived mycotoxins associated with food spoilage and are known to cause liver and kidney damage in animals [74,75]. Bioassay results showed that these three compounds revealed strong to moderate antibacterial activity against *S. aureus* and MRSA (Table 3). The biosynthesis of xanthomegnin has been studied previously in *P. freii* and a 6-MSAS PKS was identified as being associated with its production [76]. Moreover, a recent genome analysis of *Aspergillus* predicted that the biosynthetic gene clusters for xanthomegnin and vioxanthin may be associated with NR and PR PKSs [77]. Our study found that F7180 was positive for NR, PR, and HR PKS genes (Figure 2, Table 2), including a PKS grouped in the fungal 6-MSAS clade, indicating *P. janthinellum* has the capacity to produce a variety of PKS-derived compounds. Interestingly, F7180 also produced pretrichodermamide C, an alkaloid which displayed moderate antimicrobial activity against *S. aureus* and MRSA (IC_50_ < 50 µM), but with no cytotoxic activity against A549 cells (Table 2). The lack of cytotoxicity should be confirmed with testing against a normal cell line such as WI-38. Pretrichodermamide C is a new epidithiodiketopiperazine alkaloid discovered in *Penicillium* sp. and which was reported to be inactive against the L5178Y mouse lymphoma cell line [78]. Genes related to alkaloid synthesis were not screened in this study. However, future work could include screening for alkaloid biosynthesis genes since alkaloids are increasingly being recognized as a class of natural products with important bioactivities.

In summary, we have examined the species diversity and biosynthetic potential of 30 terrestrial and marine fungi isolates from St. John’s Island, Singapore, and tested their extracts for biological activities. More than half of the fungi samples (60%) had at least one PKS or NRPS gene, indicating their genetic potential for the synthesis of secondary metabolites. Our genetic screening campaign revealed various classes of fungal PKSs which could prove to be useful for comparison with fungal communities from similar environments. Bioassay-guided fractionation of culture extracts from two prioritized fungal strains resulted in the isolation of seven known compounds exhibiting varied levels of antimicrobial and cytotoxic activities. To resolve the knowledge gap between BGC identification and compound isolation, further studies could examine the relationship between the PKS/NRPS genes and the respective bioactive compounds. Elucidation of the full BGCs and biochemical characterization of the pathways would be crucial in understanding how these genes and products are functionally related.

## 4. Materials and Methods

### 4.1. Sampling and Isolation of Fungal Strains

Samples from terrestrial soil and marine sediment and macroinvertebrates were collected from St. John’s Island, Singapore (Table S3). Isolation of endophytic fungi from terrestrial plant samples was done following the procedures described by Talukdar et al. [79] while those from soil and marine sediment samples were isolated following the methods described by Montoya-Castrillón et al. [80] and Keeler et al. [81], respectively. The isolation of fungal strains from the collected samples was performed within 24 h via serial dilutions and plating on glucose, yeast extract, sea salts (GYS) or *potato dextrose agar* (PDA) plates supplemented with chloramphenicol (80 µg/mL) and incubation of the plates at 24 °C for 3–7 days. Each growing fungal culture was checked for purity and transferred to another GYS/PDA agar plate by the hyphal tip method and preserved in the Natural Product Library (NPL), Singapore Institute of Food and Biotechnology Innovation, Singapore [82]. The 30 strains of fungi (13 terrestrials and 17 marine strains) were recovered and subjected to screening for biosynthetic and antimicrobial potential. Terrestrial fungal strains were grown on malt extract agar (MEA), while the marine strains were grown on GYS agar (10 g/L glucose, 1 g/L yeast extract, and 40 g/L sea salts). Cultures were maintained at 24 °C for approximately 5 days before isolation of genomic DNA or as inoculum for batch liquid culture.

### 4.2. DNA Extraction and Identification of Fungi by ITS Sequencing

Genomic DNA was extracted using the *MagListo*™ 5 M Genomic DNA Extraction Kit (Bioneer, Republic of Korea) following manufacturers’ instructions. Mycelia were harvested into 2-mL screw-capped tubes containing 0.5 mm zirconia/silica beads (Biospec Products, Bartlesville, USA). After addition of the lysis buffer, RNase A, and Proteinase K solutions, the tubes were fixed onto a bead beater (1600 MiniG^®^, SPEX SamplePrep, Metuchen, NJ, USA) and homogenized for 25 s and finally the genomic DNA was eluted by a low salt elution buffer. DNA quality and quantity were assessed with a NanoDrop Spectrophotometer (NanoDrop Technologies, Thermo Fisher Scientific, Waltham, MA, USA). For identification of isolated fungi, internal transcribed spacer region 2 (ITS 2) was amplified using primers ITS86F (as forward primer) and ITS4 (as reverse primer) [83] (Table S4). PCR reactions for ITS2 region were carried out in 20 µL reaction volumes that contained 2× KAPA HiFi HotStart ReadyMix (KAPA Biosystems, Wilmington, MA, USA), 0.5 µM ITS primers, and 1 µL of genomic DNA. The PCR conditions were as follows: initial denaturation at 95 °C for 5 min, followed by 30 cycles at 95 °C for 30 s, 55 °C for 30 s, 72 °C for 30 s, and a final extension step at 72 °C for 10 min, before holding at 12 °C. A negative control was included in which DNA was replaced with sterile distilled water. Amplified PCR products were purified using a DNA purification kit (iNtRON Biotechnology, Republic of Korea) according to the manufacturer’s instructions. The ITS gene sequence was determined by BigDye Terminator v3.1 cycle sequencing (Applied Biosystems, Waltham, CA, USA). Each sequence was analyzed using BLASTN against the NCBI Nucleotide database to determine the identity of strains or their nearest match [84].

### 4.3. Amplification, Cloning, and Sequencing of the PKS/NRPS Gene Regions

Degenerate primer PCR is an efficient method for identification of novel PKS and NRPS genes from fungi whose genome sequences are not available [85]. Hence, we performed amplicon sequencing to identify the biosynthetic genes involved in the biosynthesis of secondary metabolites. Type I PKS genes were amplified using the following degenerate primer pairs designed to target the ketosynthase domain of PKS: primers LC1/LC2c for non-reducing (NR) class of KS domains [38], LC3/LC5c for partially reducing (PR) class of KS domains [38] and KS3/KS4 for highly reducing (HR) class of KS domains [39] (Table S4). Primers AUG003/AUG007, designed to recognize the NRPS consensus cores of adenylation (A3) and thiolation (T) domains respectively, were used for amplification of NRPS [8,40] (Table S4). The PCR products spans a T domain connected to an A domain, exhibiting a specific set of conserved motifs representing a fingerprint that is exclusively found in peptide synthetases [86]. PCR products of the expected sizes were excised from agarose gels, purified, and cloned into pGEM^®^-T Easy Vector (Promega, Madison, WI, USA) for sequencing. Purified DNA fragments with a low quantity of products were re-amplified with the same primer pairs before cloning into pGEM-T Easy vector. Sequencing was performed with M13 forward and reverse primers situated on the plasmid. Nucleotide sequences of the cloned fragments were deposited in GenBank. Their accession numbers are provided in Table S1.

### 4.4. Sequence and Phylogenetic Analyses of PKS and NRPS Fragments

PKS and NRPS gene sequences were analyzed for a similarity to known sequences in NCBI using BLASTN and BLASTX [84]. For construction of phylogenetic trees, nucleotide sequences were translated into amino acids using the ExPASy translation tool [87], aligned using CLUSTALW [88], and imported into MEGA 7 software [89]. Reference sequences for clade designation were assigned according to a previous study [43].

### 4.5. Shake-Flask Fermentation and Metabolites Extraction

Fungal strains were grown in 50 mL CF02LB liquid media (Maltose 10 g/L; Yeast extract 4 g/L; Glucose 20 g/L; Oatmeal 20 g/L) in 250-mL Erlenmeyer flasks in a shaking incubator (INFORS HT Multitron) at 200 rpm at 24 °C for 14 days. Three fungal agar plugs (4 mm diameter) were cut using sterile plastic drinking straws from the peripheries of 7-day-old cultures for the fast-growing isolates and 14-day-old cultures for the slow-growing isolates and aseptically transferred to the media. After 9 days of fermentation, cultures were freeze-dried and extracted with equal volume (50 mL) of methanol (MeOH) by overnight shaking (200 rpm, 24 °C), followed by filtration through a Whatman #4 filter paper (20–25 μm pore size; 1004–185; Whatman) and dried using a vacuum rotary evaporator (MiVac Quattro Concentrator). The dry crude extracts were stored at 4 °C until when required for bioassays and chemical analyses.

### 4.6. Antimicrobial Testing of the Crude Extracts, Fractions and Purified Compounds against S. aureus, Methicillin-Resistant S. aureus, E. coli and C. albicans

Dried extracts were resuspended in MeOH to obtain 20 mg/mL sample concentration followed by sonication for complete solubilizing. Ten microliters (10 µL) of the extracts per well were added to 96-well plates followed by drying via centrifugal vacuum concentrator. Upon drying, 100 µL of 10% DMSO was added to each well to obtain a sample concentration of 2 mg/mL. Ten microliters (10 µL) were subsequently used for the antimicrobial bioassays where 90 µL of the respective microbial cultures were added to each sample, leading to a final extract concentration of 200 µg/mL. For preliminary screening, extracts from all 30 fungi were evaluated for antimicrobial activities using the highest concentration of 200 µg/mL. Extracts that showed an average growth inhibition of ≥50% were then subjected to further bioassays using the serial dilution method [90] with two-fold dilutions, allowing for the determination of the half maximal inhibitory concentrations (IC_50_).

For the antibacterial bioassays, the test microorganisms *S. aureus*, Methicillin-resistant *S. aureus* (MRSA) and *E. coli* were plated on Mueller-Hinton II agar (Merck, Germany) and incubated overnight at 37 °C. Colonies were added to a glass tube containing 0.85% saline solution until the turbidity was equivalent to 0.5 McFarland Standard (approximate cell density 1.5 × 10^8^ CFU/mL). Cell suspensions were then diluted in MH2 liquid media to obtain a final cell density of 5 × 10^5^ CFU/mL. Ninety microliters (90 µL) of cell suspensions were added to 10 µL of extracts in the 96-well bioassay plates and incubated at 37 °C for 20 h. For each extract tested, three replicates were used. The optical density (OD) of each well was measured using a Hidex Sense microplate reader (Hidex, Finland) at 600 nm. Percentage growth inhibition was calculated according to the following formula with the quality of the bioassay assessed based on the Z-prime analysis [91]. A Z-prime of ≥0.7 indicates the robustness of the high throughput screening data. Gentamicin was used as the standard control against *S. aureus* and *E. coli* while vancomycin was used as the standard control in the MRSA assay.

For the *C. albicans* screen, a single colony from freshly streaked *C. albicans* on Sabouraud dextrose agar (SDA) was inoculated into 25-mL of Sabouraud liquid medium (SLM) and incubated at 35 °C with shaking at 150 rpm for 18 hrs. Subsequently, 1% of the culture (250 µL) was prepared with SLM and incubated at 35 °C with shaking for 6 h. OD_600_ was monitored until it reached approximately 0.8. Cell density (D) of the culture was calculated using the following equation: D = [(30 × OD_600_) − 8] × 10^6^ CFU/mL. Final cell densities were adjusted to 2.5 × 10^5^ CFU/mL and inoculated as 90 µL suspensions into 96-well plates. Similar to the antibacterial assays, upon addition of 10 µL of extracts to the cell suspensions, the plates were incubated at 37 °C for 20 h prior to OD readings. For each extract tested, three technical replicates were used. Amphotericin B was used as the antifungal standard control in *C. albicans* screen.

### 4.7. Cytotoxicity Assay

The cytotoxic effect of the isolated compounds on the A549 human lung carcinoma cell line was determined as described previously [92]. A549 cells at a concentration of 3.3 × 10^4^ cells/mL in Dulbecco’s Modified Eagle Medium (DMEM, Gibco, Life Technologies, Bleiswijk, The Netherlands) were treated with isolated compounds at concentrations ranging from 0.1 μM to 200 μM and incubated for 72 h at 37 °C in 5% CO_2_ and 95% relative humidity. Cytotoxic effect of the compounds was measured after the addition of PrestoBlue™ cell viability reagent (Thermo Fisher Scientific, USA) and incubation for 2 h by reading of the plates’ fluorescence at an excitation wavelength of 560 nm and emission wavelength of 590 nm using the Tecan Infinite M1000 Pro reader. Puromycin served as the standard control.

### 4.8. Large Scale Fermentation and Extraction of Bioactive Compounds

Fungal strains F4335 and F7180 were subcultured in malt extract agar (MEA, Oxoid, UK) for 7 days at 24 °C. Three agar plugs of 5 mm diameter from the culture plate were then used to inoculate into 4 × 250 mL Erlenmeyer flasks containing 50 mL of seed media [yeast extract 4 g/L, malt extract 10 g/L, glucose 4 g/L, pH5.5] and incubated for 5 days at 24 °C, with shaking at 200 rpm. An amount of 2.5 mL of the homogenized seed culture was used to inoculate 50 mL of fermented media in 250 mL Erlenmeyer flasks to obtain a total large-scale fermentation of 2 L and 4 L for strains F7180 and F4335, respectively. The cultures were incubated at 24 °C in shaking incubators at 200 rpm. At the end of the incubation period, the cultures were frozen overnight at −80 °C and freeze-dried in a vacuum freeze-dryer to expel all moisture followed by extraction overnight with MeOH and dichloromethane (DCM) for strains F4335 and F7180, respectively. The extract mixture was subjected to filtration using filter paper (Whatman No. 4) to separate the supernatant from the insoluble media components and mycelial residues. The filtrate was dried using a rotary evaporator to generate dried crude extracts.

### 4.9. General Chemistry Experimental Procedure

All nuclear magnetic resonance (NMR) spectra were recorded using Bruker DRX-400 NMR spectrometer with Cryoprobe. The HR-ESI-MS spectra were acquired on Agilent UHPLC 1290 Infinity coupled to Agilent 6540 accurate-mass quadrupole time-of-flight (QTOF) mass spectrometer equipped with a splitter and an ESI source. The small-scale crude extracts fractionation for assay testing were prepared at a concentration of 20 mg/mL and were fractionated on an Agilent Poroshell SB-C18 4.6 × 75 mm, 2.7 μm column at a flow rate of 2 mL/min, under a standard gradient condition of 0.1% formic acid (HCOOH) in water (solvent A) and 0.1% HCOOH in acetonitrile (solvent B) over 14 min using Agilent UHPLC 1290 Infinity coupled to Agilent 6540 accurate-mass quadrupole time-of-flight (QTOF) mass spectrometer system. The fractions were collected and dried in a 96-well microtiter plate using centrifugal evaporator. The dried fractions were tested against *S. aureus*, MRSA, and *C. albicans* and the active fractions were analysed by LCMS.

The large-scale compounds isolation of F4335 and F7180 were separated on an Agilent Prep C18 column (100 × 30 mm) by gradient elution with a mixture of 0.1% HCOOH in water (solvent A) and 0.1% HCOOH in acetonitrile (solvent B).

### 4.10. Purification and Chemical Characterization of Isolated Compounds

F4335: Approximately 1 g of dried MeOH crude extracts were re-dissolved in MeOH and subjected to C-18 reversed-phase preparative HPLC for fractionation (solvent A: H_2_O + 0.1% HCOOH, solvent B: acetonitrile + 0.1% HCOOH; flow rate: 30 mL/min, gradient conditions: 5% solvent B isocratic for 5 min, followed by 5% to 20% of solvent B over 10 min, then from 20% to 60% solvent B over 50 min, and from 60% to 100% solvent B in 30 min, and finally isocratic at 100% solvent B for 15 min). Fractions eluted at 23.2 min, 32.2 min, and 52.1 min were identified as penilumamide D (**1**, 2.5 mg) [93], penilumamide (**2**, 4.2 mg) [73], and penilumamide B (**3**, 3.1 mg) [93], respectively. The isolated compounds were identified based on the LCMS and by comparison with the reported NMR data.

F7180: The freeze-dried biomass was extracted using DCM and the solvent was evaporated to dryness using rotary evaporator. Approximate 1 g of dried DCM crude extracts were re-dissolved in DMSO solvent and subjected to C-18 reversed-phase preparative HPLC for fractionation (solvent A: H_2_O + 0.1% HCOOH, solvent B: acetonitrile + 0.1% HCOOH; flow rate: 30 mL/min, gradient conditions: 20% solvent B isocratic for 5 min, followed by 20% to 50% solvent B over 15 min, then from 50% to 80% solvent B over 60 min, and subsequently from 80% to 100% in 10 min, and finally isocratic at 100% solvent B for 15 min). Fractions eluted at 8.0 min, 23.5 min, 29.6 min, and 37.2 min were identified as pretrichodermamide C (**4**, 1.3 mg) [78], xanthomegnin (**5**, 2.8 mg) [94,95,96], viomellein (**6**, 1 mg) [97,98], and vioxanthin (**7**, 0.4 mg) [99], respectively. The isolated compounds were identified based on the LC-MS and comparison with the reported NMR data.

## 5. Conclusions

In this study, diversity, biosynthetic and biological potential of ascomycetes fungi isolated from St. John’s Island, Singapore has been investigated, and all the identified fungal strains were affiliated to the phylum *Ascomycota* and formed six groups according to their Order with *Aspergillus*, *Pestalotiopsis*, and *Purpureocillium* being the most prevalent genera. Screening of the fungal strains on the basis of their reduction capabilities has revealed that more than half of the fungal stains (60%) had at least one PKS or NRPS biosynthetic genes, giving an indication of their genetic potential for the synthesis of this group of secondary metabolites. Methanolic extracts generated from the 30 identified fungal strains have revealed various levels of antimicrobial and cytotoxic activities. Two fungal strains, F4335 and F7180 revealed promising antimicrobial activity and were progressed to large-scale fermentation. Bioassay-guided compound isolation studies led to the isolation of seven bioactive compounds: penilumamide A, D, and E, xanthomegnin, viomellein, pretrichodermamide C, and vioxantin from the two fungal isolates. This study provides valuable insights into the diversity and biosynthetic potential of fungi isolated from this unique and largely untapped habitat and could pave the way for a more comprehensive study which could lead to the discovery of fungal isolates capable of producing molecules with interesting biological activities.

## Figures and Tables

**Table 1:** PKS and NRPS identified with the degenerate primers. BLASTX matches with the highest scoring annotated protein sequence from NCBI are shown.

Gene	ID	Fungi Species	BLASTX Match	Accession No.	Identities (%)
NR PKS	4323	*Trichoderma harzianum*	beta-ketoacyl synthase domain-containing protein [*Trichoderma harzianum*]	KKP03797.1	232/262 (89)
NR PKS	4332	*Pestalotiopsis vismiae*	polyketide synthase [*Pestalotiopsis microspora*]	APX43975.1	231/263 (88)
NR PKS	4333	*Colletotrichum gloeosporioides*	polyketide synthase [*Colletotrichum gloeosporioides* Nara gc5]	XP_007287024.1	238/240 (99)
NR PKS	4361	*Curvularia geniculata*	polyketide synthetase ClPKS18 [*Curvularia lunata* CX-3]	AUI38916.1	236/240 (98)
NR PKS	4366	*Aspergillus nomius*	putative WA type ketosynthase domain 2 [*Aspergillus parasiticus*]	CAB44699.1	239/240 (99)
NR PKS	4619	*Stachybotrys parvispora*	polyketide synthase [*Pseudocercospora griseola*]	AGI04994.1	226/234 (97)
NR PKS	4625	*Curvularia lunata*	polyketide synthase [*Aspergillus terreus*]	BAB88689.1	221/240 (92)
NR PKS	4710	*Aspergillus polyporicola*	polyketide synthase [*Aspergillus terreus*]	BAB88689.1	220/240 (92)
NR PKS	6365	*Aspergillus fumigatus*	putative WA type ketosynthase domain 2 [*Aspergillus parasiticus*]	CAB44699.1	215/240 (90)
NR PKS	7161	*Cladosporium tenuissimum*	putative non-reducing polyketide synthase 1 [*Ramularia collo-cygni*]	ADZ14597.1	227/241 (94)
NR PKS	7163	*Pestalotiopsis vismiae*	hypothetical protein PFICI_10824 [*Pestalotiopsis fici* W106-1]	XP_007837596.1	232/244 (95)
NR PKS	7180	*Penicillium janthinellum*	polyketide synthase [*Penicillium oxalicum*]	AMD78094.1	236/240 (98)
PR PKS	4335	*Aspergillus flavipes*	hypothetical protein EPUS_00492 [*Endocarpon pusillum* Z07020]	XP_024705167.1	174/227 (77)
PR PKS	4352	*Chordomyces antarcticum*	polyketide synthase [*Fusarium graminearum*]	AAS57290.1	184/227 (81)
PR PKS	4619	*Stachybotrys parvispora*	polyketide synthase, putative [*Aspergillus flavus* NRRL3357]	XP_002385535.1	219/228 (96)
PR PKS	4624	*Eutypella scoparia*	polyketide synthase [*Aspergillus terreus*]	AFK08433.1	189/228 (83)
PR PKS	4710	*Aspergillus polyporicola*	polyketide synthase [*Aspergillus terreus*]	AFK08433.1	155/228 (68)
PR PKS	4914	*Trichoderma virens*	putative PKS-NRPS protein [*Trichoderma virens* Gv29-8]	XP_013957207.1	226/249 (91)
PR PKS	7165	*Acremonium sp.*	putative PKS-NRPS protein [*Trichoderma virens* Gv29-8]	XP_013957207.1	205/227 (90)
PR PKS	7180	*Penicillium janthinellum*	polyketide synthase [*Aspergillus terreus*]	AFK08433.1	152/187 (81)
HR PKS	4332	*Pestalotiopsis vismiae*	polyketide synthase [*Pestalotiopsis microspora*]	AFK08433.1	200/209 (96)
HR PKS	4333	*Colletotrichum gloeosporioides*	capsular polysaccharide biosynthesis fatty acid synthase [*Colletotrichum higginsianum*]	AFK08433.1	176/209 (84)
HR PKS	7180	*Penicillium janthinellum*	reducing polyketide synthase [*Penicillium janthinellum*]	XP_013957207.1	145/145 (100)
NRPS	4323	*Trichoderma harzianum*	hypothetical protein PISL3812_08019 [*Talaromyces islandicus*]	CRG90971.1	284/425 (67)
NRPS	4339	*Chordomyces antarcticum*	Nonribosomal peptide synthetase 4-like protein 2 [*Colletotrichum chlorophyti*]	AFK08433.1	321/404 (79)
NRPS	4352	*Chordomyces antarcticum*	Nonribosomal peptide synthetase 4-like protein 2 [*Colletotrichum chlorophyti*]	APX43987.1	319/404 (79)
NRPS	4710	*Aspergillus polyporicola*	Nonribosomal Peptide Synthase (NRPS) [*Pseudogymnoascus* sp. 23342-1-I1]	CCF35219.1	329/432(76)
NRPS	6365	*Aspergillus fumigatus*	nonribosomal peptide synthase [*Aspergillus fumigatus* Z5]	ADY75760.1	426/431 (99)

**Table 2:** Summary of biosynthetic and antimicrobial potential in 30 strains of ascomycetes from St. John’s Island, Singapore. Antimicrobial activity is defined as the ability of crude extracts to inhibit the growth of the test microorganisms by more than 50%. T, terrestrial; M, marine.

Strain ID	Identified Genus/Species	Habitat	Signature Biosynthetic Genes				Antimicrobial Activity		
			PKS (NR)	PKS (PR)	PKS (HR)	NRPS	SA	MRSA	CA
4323	*Trichoderma harzianum*	T	+	−	−	+	−	−	−
4332	*Pestalotiopsis vismiae*	T	+	−	+	−	−	−	−
4333	*Colletotrichum gloeosporioides*	T	+	−	+	−	−	−	−
4335	*Aspergillus flavipes*	T	−	+	−	−	+	+	−
4339	*Chordomyces antarcticum*	T	−	−	−	+	−	−	−
4352	*Chordomyces antarcticum*	T	−	+	−	+	−	−	−
4361	*Curvularia geniculata*	T	+	−	−	−	−	−	−
4366	*Aspergillus nomius*	T	+	−	−	−	+	−	−
4374	*Acremonium pilosum*	T	−	−	−	−	−	−	−
4608	*Westerdykella dispersa*	M	−	−	−	−	−	−	−
4619	*Stachybotrys parvispora*	M	+	+	−	−	−	−	−
4624	*Eutypella scoparia*	M	−	+	−	−	−	−	−
4625	*Curvularia lunata*	M	+	−	−	−	−	−	−
4627	*Purpureocillium lilacinum*	M	−	−	−	−	−	−	−
4632	*Phoma* sp.	M	−	−	−	−	−	−	−
4708	*Fusarium solani*	T	−	−	−	−	−	−	−
4710	*Aspergillus polyporicola*	T	+	+	−	+	−	−	−
4908	*Pestalotiopsis microspora*	T	−	−	−	−	−	−	−
4914	*Trichoderma virens*	T	−	+	−	−	−	−	−
6365	*Aspergillus fumigatus*	M	+	−	−	+	−	−	−
7160	*Purpureocillium lilacinum*	M	−	−	−	−	−	−	−
7161	*Cladosporium tenuissimum*	M	+	−	−	−	−	−	−
7163	*Pestalotiopsis vismiae*	M	+	−	−	−	−	−	−
7165	*Acremonium* sp.	M	−	+	−	−	−	−	−
7169	*Mariannaea elegans*	M	−	−	−	−	−	−	−
7172	*Cladosporium cladosporioides*	M	−	−	−	−	−	−	−
7178	*Gliomastix polychroma*	M	−	−	−	−	−	−	−
7180	*Penicillium janthinellum*	M	+	+	+	−	+	+	+
7374	*Ascotricha sinuosa*	M	−	−	−	−	−	−	−
7602	*Purpureocillium lilacinum*	M	−	−	−	−	−	−	−

Signature biosynthetic (+) = present (−) = absent; Antimicrobial activity (+) = active i.e., average percent inhibition ≥ 50 (−) = inactive i.e., average percent inhibition < 50; Habitat (T) = terrestrial, (M) = marine. SA (*S. aureus*), MRSA (Methicillin-resistant *Staphylococcus aureus*), CA (*C. albicans*).

**Table 3:** Bioactivity of compounds isolated from fungal strains F4335 (*Aspergillus flavipes*) and F7180 (*Penicillium janthinellum*) against SA (*Staphylococcus aureus* ATCC 25923), MRSA (Methicillin-resistant *Staphylococcus aureus* ATCC 33591), EC (*Escherichia coli* ATCC 25922), CA (*C. albicans* ATCC 10231) and A549 (human lung carcinoma cells ATCC CCL-185).

Strain ID	Compound ID	Compound Name	IC_50_ (μM)				
			SA	MRSA	EC	CA	A549
F4335	N2638	Penilumamide D	>200	>200	>200	>200	>200
F4335	N1260	Penilumamide A	88.0	>200	>200	>200	100.0
F4335	N2639	Penilumamide B	>200	>200	>200	>200	113.0
F7180	N1134	Xanthomegnin	19.2	35.3	>200	18.6	65.6
F7180	N1135	Viomellein	3.2	2.9	>100	17.9	42.0
F7180	N2669	Pretrichodermamide C	34.7	41.6	>200	>200	>200
F7180	N2690	Vioxanthin	3.0	1.6	>200	36.4	77.1
-	-	Gentamicin	0.22	-	0.67	-	-
-	-	Vancomycin	-	1.5	-	-	-
-	-	Amphotericin B	-	-	-	0.14	-
-	-	Puromycin	-	-	-	-	0.25

## Data Availability

Not applicable.

## Supplementary Materials

The supporting information can be downloaded at: https://www.mdpi.com/article/10.3390/ijms24021033/s1.
